# Feasibility of a cluster randomized controlled trial on the effectiveness of peer–led health education interventions to increase uptake of retinal examination for diabetic retinopathy in Kirinyaga, Kenya: a pilot trial

**DOI:** 10.1186/s40814-020-00644-8

**Published:** 2020-07-16

**Authors:** Nyawira Mwangi, Covadonga Bascaran, Mark Ng’ang’a, Jacqueline Ramke, Mathew Kipturgo, Stephen Gichuhi, Min Kim, David Macleod, Consuela Moorman, David Muraguri, Esbon Gakuo, Lawrence Muthami, Allen Foster

**Affiliations:** 1 Kenya Medical Training College, Nairobi, Kenya; 2 London School of Hygiene and Tropical Medicine, London, UK; 3 Kerugoya County Referral Hospital, Kerugoya, Kenya; 4 University of Nairobi, Nairobi, Kenya; 5 Oxford Universities NHS Trust, Oxford, UK; 6 Kenya Medical Research Institute, Nairobi, Kenya

**Keywords:** Diabetes, Diabetic retinopathy, Peer support, Kenya, Pilot, Cluster randomised controlled trial

## Abstract

**Background:**

People living with diabetes can reduce their risk of vision loss from diabetic retinopathy by attending screening, which enables early detection and timely treatment. The aim of this pilot trial was to assess the feasibility of a full-scale cluster randomized controlled trial of an intervention to increase uptake of retinal examination in this population, as delivered within existing community-based diabetes support groups (DSGs).

**Methods:**

All 16 DSGs in Kirinyaga county were invited to participate in the study. The first two groups recruited took part in the pilot trial. DSG members who met the eligibility criteria were recruited before the groups that were randomized to the two arms. In the intervention group, two peer educators were trained to deliver monthly DSG-based eye health education and individual telephone reminders to attend screening. The control group continued with usual DSG practice which is monthly meetings without eye health education. The recruitment team and outcome assessors were masked to the allocation. We documented the study processes to ascertain the feasibility, acceptability, and potential effectiveness of the intervention. Feasibility was assessed in terms of clarity of study procedures, recruitment and retention rates, level of acceptability, and rates of uptake of eye examination. We set the target feasibility criteria for continuation to the main study to be recruitment of 50 participants in the trial, 80% monthly follow-up rates for individuals, and no attrition of clusters.

**Results:**

Of the 122 DSG members who were assessed for eligibility, 104 were recruited and followed up: 51 (intervention) and 53 (control) arm. The study procedures were well understood and easy to apply. We learnt the DSG meeting days were the best opportunities for recruitment. The study had a high acceptance rate (100% for clusters, 95% for participants) and high follow-up and retention rate (100% of those recruited). All clusters and participants were analysed. We observed that the rate of incidence of eye exam was about 6 times higher in the intervention arm as compared to the control arm. No adverse unexpected events were reported in either arm.

**Conclusions:**

The study is feasible and acceptable in the study population. The results support the development of a full-scale cluster RCT, as the success criteria for the pilot were met.

**Trial registration:**

Pan African Clinical Trials Registry PACTR201707002430195 Registered on 25 July 2017.

## Background

The long-term complications of diabetes, such as diabetic retinopathy (DR), are a threat to health among people living with diabetes (PLWD). DR is a growing concern in global health epidemiology due to the high proportion of DR that remains undetected. Vision loss from DR can be prevented through regular retinal screening (hereafter referred to as “screening”) and timely treatment [1–3] There is notable geographic variation in the incidence and visual impairment burden of DR, both within and between countries, reflecting variation in access to health care [4–8]. Services for DR prioritize early detection, metabolic control, regular monitoring, and timely treatment. Access to these services is a significant challenge due to demand side barriers (such as low awareness of the need for services among PLWD) and supply side barriers (such as availability of clinical guidelines or screening services) [9]. There is need for better evidence and patient empowerment to address the demand side barriers, as well as health system strengthening to address supply-side barriers [9–11].

In the *Global action plan for the prevention and control on non-communicable diseases (NCDs) 2013–2020,* the World Health Organization (WHO) highlighted the need to empower people with NCDs to seek early detection, and to provide them with appropriate education, incentives, and tools for self-management [12]. The peer support model has been used in diabetes and other chronic conditions to improve social support and self-management, with positive outcomes in other countries [13–17]. Traditionally, peer support model has not been used in diabetes eye health services and subsequently, there is a knowledge gap regarding its effectiveness to reduce vision loss from diabetic retinopathy. Leveraging on peer support in a clinical or community setting might be a potential enabler for the adoption of healthy behaviours, such as screening.

Clinical guidelines for the management of DR target were to have a 100% attendance of PLWD at annual screening [18]. Our research group has previously reported that PLWD in three counties of Kenya have low attendance to annual screening, which is the frequency recommended in this setting [19]. This is consistent with findings that uptake of DR screening is low in many parts of the world, but more so where access to health care is generally limited [9]. To address this deficit, the Uptake of Retinal Examination in Diabetes (DURE) trial [20] aims to test the effectiveness of peer support in increasing the uptake of retinal examination among members of diabetes support groups (DSGs). Diabetes support groups are volunteer social groups of PLWD in which peers provide mutual support for improving diabetes care. The support may include information and skills for self-management, as well as emotional support. Given this objective, DSG members are likely to be health conscious and interested in adopting healthy behaviours. This intervention in this study is based on the self-efficacy theory [21] and is targeted to PLWD who are already members of support groups and have not had screening in the previous 12 months or longer. Screening in this setting involves a visual acuity test and a retinal examination through a dilated pupil [18, 22].

The study setting is a rural county whose inhabitants are mainly small scale farmers. The DSGs are spread over the 1200 km^2^ area of the county. Undertaking the DURE study raises important practical concerns. In this pilot study, our aim was to gain experience in delivering the intervention and to assess if the DURE cluster randomized clinical trial (cRCT) is feasible by (1) testing clarity and ease of study procedures for enrolment and data collection, (2) determining the potential for participant recruitment and retention, (3) assessing the acceptability of the intervention, by considering the level of adoption of the study interventions by different actors, and (4) documenting an interim measure of the effectiveness of the intervention on the uptake of screening. Our hypothesis was that it is feasible to conduct the DURE study. We set the target feasibility criteria for continuation to the main study to be recruitment of 50 participants in each cluster, at least 80% follow-up rate of participants in each month of the trial and no attrition of clusters. The 90-day duration of the pilot trial was considered sufficient for these feasibility objectives, while the main trial will take 6 months.

## Methods

### Study setting

The demographic and health statistics of Kirinyaga county are highlighted in Table 1. The study intervention was developed following a health system assessment for diabetes and diabetic retinopathy in three counties of Kenya [23] conducted by our research team, which identified gaps in access to services for DR, as well as the need for health system strengthening. We found that only 7% of PLWD in this county had a DR screening exam in the preceding 12 months. The main barriers to access are lack of referral from diabetes services, lack of knowledge of diabetes eye complications among PLWD, and the belief that a screening exam is only necessary once ocular symptoms develop.

**Table 1:** Demographic and health statistics—Kirinyaga county

Parameter	Kirinyaga county	Kenya
Total population (estimates based on 2009 census) [24]	595,379	48.5million
Females	50%	50%
Age > 18 years	409,995	22,005,235
Urban population	16%	29.9%
No of people with diabetes (2%) [25]	8,185	440,104
No of people needing an annual eye exam	8,185	440,104
No of eye care facilities	1	112
No of ophthalmologists	1	115
No of ophthalmic clinical officers	3	300
No of ophthalmic nurses	0	200

An estimated 25–30% of the PLWD in the county are regular members of DSGs (with a registration number) while another 20% of PLWD attend some DSG meetings even though they are not members. Members are recruited by peer group leaders and community health volunteers as they give group health talks at community meetings, churches, outreach camps, and diabetes clinics in health facilities. As membership is entirely voluntary, the distribution of members by demographic parameters in different groups varies. All groups are under the Kenya Defeat Diabetes Association, which provides them with equipment for use within the group (such as a glucometer and a blood pressure machines). The association also trains peer supporters and DSG leaders.

DSGs hold routine monthly meetings at a dedicated time and location in the community. Eighty percent of the members attend at least two thirds of the meetings annually. The meetings are held in the morning, starting between 8 and 9 am and last 2–3 h. Each member’s fasting blood sugar, blood pressure, and weight are recorded. The group then shares a light meal. The cost of the blood sugar test strips and meal is met by a contribution of Kenya shillings 100 (the equivalent of 1$ dollar) per PLWD attending the meeting. The other activities in the meeting include group health talks delivered by peer supporters, informal discussions among PLWD, planning for advocacy, and awareness-raising activities. Record of these activities is captured in attendance registers and minutes of the meeting.

### Sample

All 16 DSGs in Kirinyaga were eligible for inclusion. We invited the DSG leaders to a meeting where we explained the objectives of the DURE study and invited all the groups to participate. The leaders then took time to discuss the study with their members before giving approval through signing consent forms. The first two DSGs to confirm willingness to participate were included in the pilot study, for simplicity, transparency, and visibility to the DSG leaders. All sixteen DSGs consented to participate; hence, the remaining 14 will participate in the main study.

We aimed to recruit at least 50 members who met the eligibility criteria (Table 2) in each DSG (average size of DSGs is 100 members). This is the same cluster size calculated for the main study, using formula for sample size calculation in cRCTs, provided by Hayes and Bennett [26]. A statistician not involved with the fieldwork conducted the sample size calculation. We also recruited two peer supporters (1 male and 1 female) who met the eligibility criteria to deliver the intervention in the intervention cluster.

**Table 2:** Eligibility criteria

Eligibility criteria for participants	Eligibility criteria for peer supporters
Inclusion criteria	Inclusion criteria
Age > 18 years	Age > 18 years
Member of a diabetes support group	Member of a diabetes support group
Will reside in the county for the next 12 months	Will reside in the county for the next 12 months
Has a mobile phone	Has a mobile phone
Willing to participate in the study	Willing to participate in the study
Has not had a screening exam in the last 12 months	Willing to be a peer educator
	Willing to commit 2 days for training
	Willing to commit many hours to peer support work
	Fluent in Kikuyu or Kiswahili
	Has had a screening exam in the preceding 12 months
**Exclusion criteria**	**Exclusion criteria**
Already attending DR screening	On treatment for DR
On treatment for DR	Has a debilitating illness
Has a debilitating illness	

### Design

The pilot study design mimics the design of the main study, being a two arm cRCT with a 1:1 ratio. A research nurse who is a local health worker recruited participants who met the eligibility criteria during a DSG meeting. The list of existing DSG members was provided by the DSG lead. Verbal consent for recruitment and follow-up was obtained from individual participants. Baseline demographic, anthropometric, and metabolic data was collected at the time of recruitment. All participants were given a study identifier card to present at the eye clinic at the time of eye exam.

Two members of the research team observed the recruitment process to identify any difficulties with participant recruitment, eligibility criteria, or completing the data collection tool. The recruitment nurse provided additional feedback on these critical components in a debriefing session after each recruitment session.

Following participant recruitment within each of the DSGs, random allocation of the intervention was through drawing of lots. A lay person not participating in the study picked one of four sealed and opaque envelopes from a container, in the presence of two members of the research team. Each envelope contained a card bearing the name of one DSG and either “intervention” or “control.” Opening the envelope revealed the arm allocation for one group, and by inference, the allocation of the other group. This allocation was copied on the envelope and stored to provide a reference trail. The remaining envelopes were destroyed. The participants in each group were followed up by a research assistant (not involved in recruitment or outcome assessment) at monthly intervals for 90 days from the first group education session, to check retention rates.

The feasibility outcomes were the achievability of recruiting 50 participants in each cluster and the viability of at least 80% follow-up rate of participants in each month of the trial. The records at Kerugoya County Hospital eye clinic were monitored daily, and the identifier cards of participants that attended screening were deposited in a specific container by the eye care team. These cards were then collected and given to the outcome assessment nurse (not involved in recruitment or follow-up). During the study, we also found that some eye care teams external to the county health services held outreach camps in the county and provided screening for some of the participants. As our team was alerted ahead of the outreach camps, we liaised with these teams and monitored the attendance of any study participants. As they used the same screening guidelines, any participants screened at the outreach site were taken to have the outcome of interest.

The recruitment nurse, research assistants, and the outcome assessors had no training in eye care and were masked to the cluster and participant allocation, to avoid contamination and bias. The eye care providers were also masked to the intervention allocation. It was not possible to mask the study participants or peer educators in the intervention arm, because the peer educators’ activities within the DSG were overt. As attendance to screening was assessed from hospital records, we concluded that the lack of masking could not incentivise over-reporting or under-reporting in either arm.

### Intervention

The DSG in the intervention arm received the study intervention combined with usual care for 90 days, while the control group received the usual standard of care alone (Table 3). The intervention was a monthly group health talk and individual monthly telephone reminders to attend eye exam, delivered by two peer educators (1 male, 1 female) collaboratively. The intended mechanism of the intervention and the key messages to be delivered in the health talk are described in the trial protocol [20]. Fidelity to the intervention and the influence of peer supporter characteristics will be assessed in the process evaluation of the main trial.

**Table 3:** Trial Interventions

Domain	Intervention group	Control group
**Usual care**	Monthly group meetings with general diabetes education talks, blood sugar, and blood pressure measurements	Monthly group meetings with general diabetes education talks, blood sugar, and blood pressure measurements
**Intervention**		
Peer supporters training	Two days training following a structured curriculum regarding diabetes eye disease, retinal screening, role of peer supporters, communication, and other aspects specified in the protocol [20]	
Group education	Monthly group education provided by trained peer supporters, with structured content on diabetic eye disease and retinal screening as specified in the protocol [20]	
Individual participant reminders	Monthly individual telephone reminder by peer supporters to participants to take a screening exam as specified in the protocol [20]	

We trained the peer educators for 2 days to deliver the intervention. They had already received previous training as peer educators (training provided by the Kenya Defeat Diabetes Association) and had about 4 years of experience with providing peer education. We also supported them during the implementation phase in the following ways: (1) Telephone calls were made by the team before and after each group session to discuss the sessions and any challenges faced by the peer leaders. (2) Practical support was provided to help organize the logistics for local program delivery, such as provision of telephone airtime and reimbursement for transport costs for peer educators. (3) The research team attended two of the three DSG meetings. Peer educators kept logs of the DSG attendance during group sessions and the individual reminders.

### Reporting and analysis

**Fig. 1:**
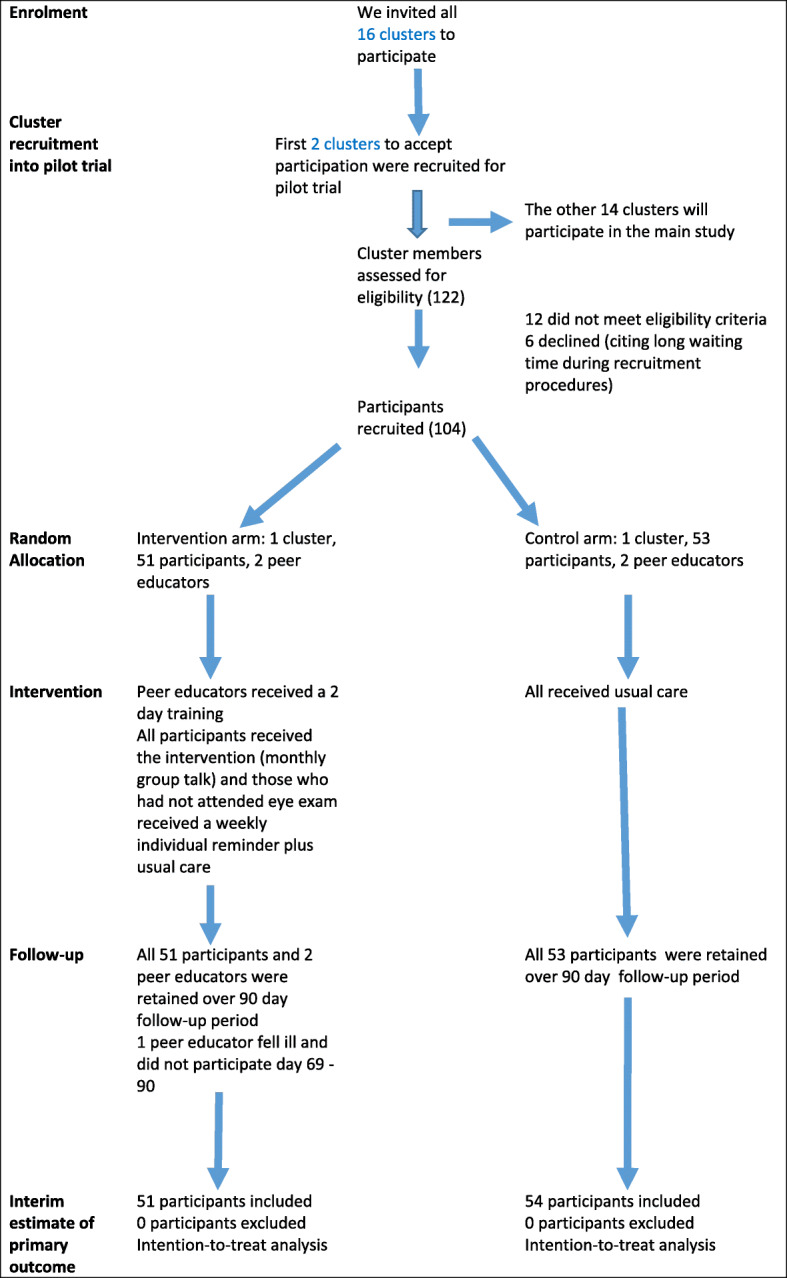
Flow diagram for the pilot study

This study is reported in accordance with the Consolidated Standards of Reporting Trials (CONSORT) guidelines for pilot studies [27]. The CONSORT flow chart (Fig. 1) and the checklist ire included. We documented recruitment procedures and rates, reasons for ineligibility and non-participation, and follow-up rates. We calculated descriptive statistics for each study arm at baseline. We also summarized survival outcomes at arm level as per intention-to-treat analysis and estimated hazard ratio for any differences. All data analysis was carried out using Stata (version 15).

## Results

The results are reported under four headings corresponding to the specific objectives of the pilot trial.

### Study procedures for enrolment and data collection

We found that the enrolment rate was high during the DSG meetings. The peer group leaders could predict which meetings would be well-attended, considering other concurrent community activities, and they also mobilised members to attend. We found it helpful to liaise with these leaders in planning for the recruitment.

The study materials were easy to carry around and work with. We did not identify any practical, ethical, or interpretation difficulties in the use of the eligibility criteria and the completion of the data collection tools. Thus, the eligibility criteria were found to be appropriate, and the data collection tools were well understood. We collected baseline data for all participants, before randomization.

The time taken to complete the data collection processes per participant exceeded the planned time. Some of the participants had difficulty finding some of the data that we needed for the purpose of follow-up, such as the telephone numbers of the next of kin, but with assistance, they were able to retrieve this from their phones or to contact other people who had the data. However, this added about 10 min per participant over the initial estimates for the recruitment process. This resulted in increased waiting time for the other persons awaiting recruitment. Learning from this, we trained three additional research nurses for the recruitment team, so that at least two of them could attend each recruitment meeting.

The data collection tools were checked for completeness at the recruitment site by a research assistant. All the data forms were also checked by the team lead for quality assurance after each recruitment meeting. Thereafter, the forms were sent to the data entry assistant where further quality checks were carried out as data was entered into a database.

We used standardised protocols for all measurements, and the descriptive data is provided in Table 4. The two arms were balanced for most characteristics except age and gender. Females and older people were over-represented in the intervention arm. This difference reflects the variation in the existing composition of the DSGs.

**Table 4:** Baseline characteristics

	Intervention (*N* = 51)	Control (*N* = 54)	Total (*N* = 104)
**Age (years)**			
Mean (SD); Median (IQR)	66.4 (10.8); 69(60-72)	58.2 (11.8); 57(57-62)	62.2 (12); 63(54.5-70)
**Sex**			
Female (%)	41 (80.4)	30 (56.6)	71 (68.3)
**Duration of diabetes (years)**			
Median(IQR)	5.0 (2–10)	5.0 (2–8)	5.0 (2–10)
**Duration of support group membership (years)**			
Median (IQR)	3.0 (1–3.5)	2.0 (1–4)	2.0 (1–4)
**Anthropometric measures**			
Body mass index (kg/m^2^)			
Male	25.0 (2)	25.3 (4.4)	25.2 (3.8)
Female	25.4 (3.9)	26.4 (3.4)	25.8 (3.7)
Waist circumference (cm)			
Male	96.5 (7.3)	96.0 (11.1)	96.1 (10)
Female	97.0 (8.9)	100.0 (8.4)	98.3 (8.8)
**Metabolic measures**			
Fasting blood sugar ( g/dl)	7.4 (2.7)	9.1 (4.5)	8.3 (3.8)
Systolic blood pressure (mmHg)	139.0 (20)	142.0 (26)	141.0 (23)
Diastolic blood pressure (mmHg)	81.0 (7.6)	78.0 (13)	80.0 (11)

### Potential for recruitment and retention

The response rate of clusters was good (both accepted to participate). All 122 participants assessed for eligibility were willing to participate, but 6 (4.9%) withdrew during the recruitment process because of long waiting time. Of the 122 assessed for eligibility, 12 (9.8%) were ineligible as they were temporary visitors (non-resident) or were going to be absent from support groups during the study period (school and employment commitments).

By conducting recruitment at well-attended routine DSG meetings, all or most of the regular DSG members had an equal chance to be recruited in the study. We were able to recruit within the anticipated time such that all the participants entered the study at time 0, and we had similar recruitment levels in both clusters. Follow-up rates were high in both arms. Both DSGs remained in the study and received the intended intervention (Fig. 1). One peer educator fell sick over part of the period, but by this time most of participants in the intervention arm already had the outcome of interest, the other peer supporter was able to carry on with the intervention. All participants were followed up for 90 days, and there was 0 loss to follow-up.

### Acceptability of the intervention

Before the start of the study, we had initial meetings with the Ophthalmic Services Unit at the national level, the Kirinyaga county director of health services, eye care providers in the county, and the national officials of Kenya Defeat Diabetes Association (KDDA) which is the umbrella body for DSGs. We obtained their buy-in for the study, and they linked us with the DSGs.

Given the recruitment rates, the follow-up rates and the attrition rates (Fig 1), the study, and the intervention were acceptable to participants and clusters. The peer supporters attended all the sessions of the 2-day training and gave the group talks as planned. For the individual telephone reminders, we found that peer educators supplemented this with face-to-face discussions with the individuals who were yet to take a retinal exam (in addition to the telephone reminders). This flexibility reflects a sense of ownership of the intervention by the peer educators.

We did not record any adverse event related to the intervention. Temporary blurring of vision after dilatation of the pupils during retinal examination (the outcome of interest) is a common undesirable effect but this was explained to the participants before examination, and none of them declined to have the examination.

The “drop-in” referral mode of patients from the DSGs for screening for DR was acceptable to both patients (who adopted it) and eye care providers (as they screened all who dropped in). The attendance to the eye clinic showed some peaks, and during these peaks, the workload in the eye clinic was significantly increased; however, all who turned up were screened. None of the participants who presented at the eye clinic had lost or forgotten the identifier card, which would suggest that the card was highly valued. Such participants would still have received screening even without the cards, and this would be captured in the eye clinic records.

### Estimating the potential effectiveness of intervention

We estimated the tentative effectiveness of the intervention in both arms, although the study was not powered for hypothesis testing. Participation rates are presented without adjustment for clustering.

**Fig. 2:**
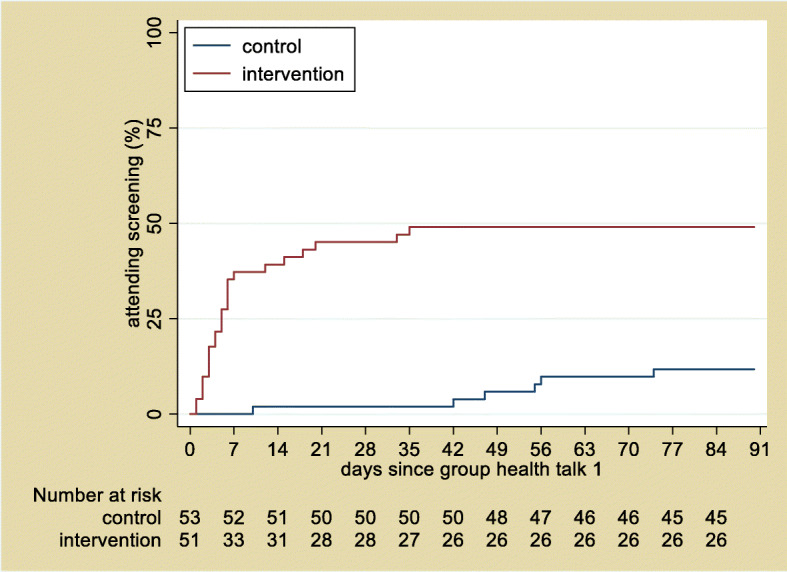
Kaplan-Meier analysis of the time from the first education session to attendance at the retinal screening (days)

The intervention arm had a substantially higher uptake of eye exam during the trial (Fig. 2). Of the 104 participants, 31(29.8%) attended screening during the trial: 25/51 (49%) in the intervention arm, as compared to 6/53 (11.3%) in the control arm.

In the intervention arm, the rate at which participants attended an eye exam was high immediately after the start of the intervention, and then it decreased (Fig. 2). The highest rates were observed between day 10 and 20 following the first group education session, even though the education sessions continued on a monthly basis. The pattern suggests that most of the benefit of the intervention occurs early in the intervention. In the control arm, the rate of eye exam was nearly constant. Even though it increased around day 50 (without any intervention), it did not reach the rate in the intervention group.

## Discussion

We are the first to report a pilot cluster RCT on the feasibility of a full-scale RCT to assess the effectiveness of a community-based DSG intervention to increase uptake of screening among PLWD. Other studies have examined the effectiveness of such community-based groups on health outcomes such as maternal, neonatal, and childhood survival [28–31].

The study has several strengths. Firstly, a mixed methods health system assessment [23] preceded the trial. This helped to identify DSGs as a community resource that was a potential channel for increasing uptake of screening. Secondly, this study targets PLWD in a rural setting who have not had screening in the last 12 months. However, we found that our participants had never had screening, which means that the people who are the most vulnerable to DR-related blindness and who need the intervention most were included. It is known that screening programs are more cost-effective in people who derive more benefit from screening [2, 7].

Thirdly, it is known that demand-side behaviour changes alone may be insufficient to change the health outcomes being addressed; therefore, health system strengthening is important before or within a trial [28, 29, 32]. Before this study, we developed and implemented national clinical guidelines for DR, and our training program for peer supporters is currently being embedded in the Kenya Defeat Diabetes Association peer support manual. Therefore, a further strength of the study is that we tested the intervention within a bigger health system strengthening context [33, 34]. Fourth, the study setting, eligibility criteria, study design, and amount of data collected in the pilot are by design, similar to what will be collected in the main study. This makes it easy to transfer the learning from the pilot to the main study.

Our findings show that the potential for recruitment and the feasibility of data collection, study implementation, and follow-up is high. There was high acceptability of the study in general and the intervention in particular, by the participants. This might be because the DSG members are already health conscious, or because of the community entry process that we followed. The top leadership of KDDA and the county director of health services introduced us to the county support group leader, who in turn introduced us to the DSGs. We had strong liaison with these stakeholders and with local health care workers which helped successful study implementation. We considered this to be important because the feasibility of the implementation and future scalability of the intervention depend on acceptability not only among the participants but also among these stakeholders in health care [35]. Further, the intervention itself also requires constant engagement with the DSGs and the participants, which may have aided the acceptability and retention of participants in the study. Of note, we did not pay the participants, they participated voluntarily.

The recruitment process was embedded in DSG meetings, which was a critical factor for efficiency in recruitment. We learnt that recruitment required more time and more research nurses than initially planned, and this will be taken into account in the main study. The pilot findings suggest that the trial should achieve high recruitment and retention. We excluded PLWD who were temporary visitors to the DSGs, as they were not likely to stay long enough to receive the intervention. Other studies have used a similar approach to avoid contamination between clusters [34]. We monitored support groups’ attendance and did not have evidence of inter-cluster migration in our study. We also learnt the necessity of liaison with mobile eye care providers from other counties who visit Kirinyaga county on eye camps, as they provided screening to this population (besides the static eye clinic at Kerugoya county referral hospital).

When interventions are implemented in real-world settings, some degree of flexible adaptation of program components occurs [35–37]. Although mobile phone interventions are useful due to their ubiquity even in this population [38], we found that face-to-face contact is valued, and that peer educators still supplemented individual telephone reminders (prescribed in the protocol) with additional face-to-face reminders to persons who had not yet taken a retinal examination. This is perhaps because of the close residential proximity of the members and the existing personal relationships between them. It also reflects that in the “real world” setting, peer support is not provided in tightly sequential or discrete categories. These flexible interactions may have contributed to the success of the intervention. The peer supporters in this study were highly experienced with peer support, having been peer supporters for a long time and having had other trainings. This may have contributed to the success of the study, and it is not known whether if we have less experienced peer supporters in the main study, we would have different findings.

In the pilot study, we observed a much greater proportion of individuals attending retinal screenings in the intervention arm than in the control arm. Given only one cluster was randomised to each arm, we cannot draw inferences from this, but it does suggest that the intervention has potential and is worth bringing forward to the full trial. Among those in the intervention arm who had eye exam, there was a striking uptake of the exam in the first two weeks of the intervention. This means the benefit of the intervention was visible within a short period. Conversely, it also means that there is risk of eye care provider fatigue if the same pattern of uptake is seen in the larger full trial. Kwaku et al. [32] have noted that such negative effects can be experienced in a clinical trial. We did not experience this in the pilot. In the full trial, we will stagger the intervention over time; hence, this challenge is unlikely to occur. As most of the PLWD only need one screening examination annually, we do not expect this to be a significant problem beyond the study.

Although the participants were aware of the risk of temporary blurring of vision during dilated eye exam, this was not a barrier to uptake of eye exam. However, since this is the first screening examination for the participants, we do not know whether it would be a barrier to future screening.

It is a good practice to consider the attributes that contribute to the scalability of interventions, even at the stage of pilot trials [39]. This pilot trial represents a step towards developing a scalable intervention because of its acceptability to participants. Acceptance by PLWD is necessary but not sufficient for scalability, since it also needs the support of service providers, administrators, and policy-makers. Based on the evidence from the pilot trial, scalability might also be feasible because of (1) the acceptability and involvement of state and non-state stakeholders who run the support groups and health services, (2) the trial is implemented within routine (pragmatic) conditions, and (3) we documented the processes involved in the trial.

The pilot study had some limitations. We only involved two DSGS in the study (out of the 16 DSGs in the county) but we considered this to be sufficient to address the issues of uncertainty in the feasibility of the study. For the main trial, we have recruited the other fourteen support groups (seven in each arm).

The first two DSGs that accepted to participate in the study were recruited in the pilot study. This convenience sample might mean that these initial findings are not generalizable to all DSGs. However, we considered that this method is helpful to demonstrate transparency to the DSG leaders and to meet the feasibility objectives. As women and older people were over-represented in the intervention arm, the extent to which the interim results of the effectiveness of the intervention can be extended to men is not clear. With the larger sample size of the main trial (700 participants), we expect more balance, but if this is not the case, we will conduct subgroup analysis.

Given that this is pragmatic trial [20], we anticipate that co-interventions may occur in the “real-world” setting of the study. We found that there were external outreach eye camps in the county during the trial period. These camps have the potential to introduce co-intervention bias, particularly if they include eye health promotion activities. However, the exposure to this co-intervention was likely balanced between the two arms, since the camps were widely publicised through community meetings and held at diverse locations across the county. In the main trial, we will identify any pre-specified or unplanned co-interventions, assess the risk of co-intervention bias, and estimate its effect on the trial outcomes.

We did not perform a process evaluation at this stage; however, this is planned as part of the main study [20], when we will have data on the primary and secondary outcomes. The process evaluation will help us understand the way the intervention worked to lead to these outcomes.

## Conclusion

The pilot study met our success criteria for the feasibility objectives. We conclude that a full trial of the intervention is feasible, and the results of this pilot will inform this full trial. The findings of this study may be relevant to other countries with a similar model of DSGs. Given the paucity of literature on implementing community-based interventions, the results of this pilot may be of interest to other researchers interested in addressing feasibility challenges in cRCT interventions targeting community groups.

## Data Availability

The data that support the findings of this study are available from the Ministry of Health, Kenya, but restrictions apply to the availability of these data, which were used under license for the current study, and so are not publicly available. Data are however available from the authors upon reasonable request and with permission of the Ministry of Health.

## Abbreviations

CONSORT: Consolidated Standards of Reporting Trials
cRCT: Cluster randomized controlled trial
DSG: Diabetes support groups
DR: Diabetic retinopathy
DURE: Uptake of retinal examination in diabetes trial
KDDA: Kenya Defeat Diabetes Association
NCDs: Non-communicable diseases
PLWD: People living with diabetes
RCT: Randomized controlled trial
